# Treatment of onychomycosis using a 1064-nm diode laser with or without topical antifungal therapy: a single-center, retrospective analysis in 56 patients

**DOI:** 10.1186/s40001-018-0340-y

**Published:** 2018-10-24

**Authors:** G. C. Weber, P. Firouzi, A. M. Baran, E. Bölke, H. Schrumpf, B. A. Buhren, B. Homey, P. A. Gerber

**Affiliations:** 1 Department of Dermatology, Heinrich-Heine-University, 40225 Düsseldorf, Germany; 2 Department of Radiation Oncology, Heinrich-Heine-University, 40225 Düsseldorf, Germany

## Abstract

**Background:**

Currently available treatment options for onychomycosis such as topical and systemic antifungals are often of limited efficacy, difficult to administer or associated with relevant side effects. Non-ablative laser therapy is proposed to represent a safe alternative without the disadvantages of drugs. Yet, to date, the efficacy of laser therapy for onychomycosis is discussed controversially. Against this background, we performed a systematic retrospective analysis of our clinical experience of 4 years of onychomycosis treatment applying a long-pulsed 1.064-nm diode laser.

**Methods:**

We retrospectively evaluated the records of 56 patients with microscopic and culturally proven onychomycosis affecting a toenail of the hallux and other toes, who had been treated with a long-pulsed 1.064-nm diode laser (FOX, A.C.R. Laser GmbH, Nuremberg) during the time period of July 2013–December 2016 with or without concomitant topical antifungals. Thereof, 27 patients received laser treatment and 29 patients received laser treatment in combination with local antifungals. We conducted a mean of 3.9 laser treatments at 2–6-week intervals. The primary endpoint of our analysis was clinical improvement; secondary endpoints were complete remission of fungal pathogens in fungal culture and in microscopy.

**Results:**

Clinical improvement was achieved in 56% of patients treated with laser only after a mean of 4.5 treatments and in 69% of patients treated with laser in combination with topical antifungals after a mean of 3.6 treatments. Cultural healing was detected in 63% of patients treated with laser only after a mean of 5.4 treatments, vs. 86% of patients treated with laser and concomitant topical antifungals after a mean of 4.8 treatments. Microscopic healing (complete healing) with the absence of fungal pathogens was achieved in 11% of patients after a mean of 4.7 treatments with laser only, vs. 21% of patients treated with laser and concomitant topical antifungals after a mean of 4 treatments. No relevant adverse effects were observed.

**Conclusions:**

The 1.064-nm diode laser is an effective and safe option for the treatment of onychomycosis. Of note, the combination with topical antifungals will increase overall treatment efficacy and reduce the time to healing. Particularly, patients with contraindications against systemic antifungals may benefit from this multimodal therapeutic approach. Our data, moreover, suggest that treatment efficacy is positively correlated with the total number of laser treatments.

## Background

Onychomycosis is one of the most prevalent nail disorders in adults affecting 4–8% of the general population worldwide [1, 2]. It is a fungal nail infection primary caused by dermatophytes [3]. The presence of previous nail trauma, diabetes mellitus and advancing age, as well as restricted peripheral circulation represent risk factors for onychomycosis [4–6]. Finally, in particular, patients with compromised immune function are at an increased risk of fungal nail diseases and are susceptible to secondary infections such as cellulitis or generalized tinea corporis. The main clinical characteristics of onychomycosis are focal parakeratosis, subungual hyperkeratosis with onycholysis (detachment of the nail plate from the nail bed) and thickening of the subungual region. Secondary superinfecting bacteria and nondermatophytic molds can give the nail plate a yellowish brown appearance. Advanced infections can then lead to total destruction of the nail plate [7, 8].

Current recommendations for the treatment of onychomycosis include the atraumatic extraction of onychomycotic nail material, e.g. via application of topical urea, and local or systemic antifungals [9]. Yet, available treatments are limited by moderate efficacy or other restraints. Topical antifungals such as amorolfine, ciclopiroxolamine or terbinafine nail lacquer often take a long time to eradicate the infection and barely penetrate the nail plate at fungicidal concentrations [10, 11]. Systemic antifungals such as fluconazole, itraconazole or terbinafine have been associated with relevant side effects such as congestive heart failure, hepatotoxicity and systemic drug interactions [4, 12, 13]. To conclude, long duration of treatment, bad compliance, severity of side effects or simply patient refusal of a systemic therapy, as well as patients not responding to treatment represent the main challenges in onychomycosis therapy.

Against this background, almost a decade ago non-ablative laser therapy was introduced as a novel treatment option for onychomycosis. In 2010, the FDA approved the use of a 1.064-nm Nd:YAG laser for this indication [14]. Since then, a broad range of different laser systems for the treatment of onychomycosis were introduced into the market [15].

Whereas the definite mode of action of non-ablative lasers for onychomycosis therapy remains unclear, one current hypothesis is the selective photothermolysis of pigments of dermatophytes with a consecutive generation of temperatures of greater than 50 °C. This heating is proposed to cause the destruction of fungal structures and thereby to eradicate the fungal infection of the nail [16–22]. An alternate hypothesis proposes an unspecific effect of tissue heating with a subsequent increase in circulation due to vasodilatation and stimulation of an immunological cure of the infection [23].

Today, laser therapy of onychomycosis is discussed controversially. Several case series, comments and studies published in the past decade report divergent results and conclusions with regard to efficacy, adverse effects and safety or even necessity of the treatment [15, 24, 25]. Of note, a systematic assessment of these published data also shows great inhomogeneity with regard to treatment protocols, endpoints, techniques, time points of analysis, and many other factors, which makes it difficult to compare results and to support drawn conclusions. Taken together, it can, however, be concluded from published data that sole laser treatment is likely less effective than pharmacological therapies. To date, analyses assessing potential synergies of laser therapy in combination with topical or systemic antifungals are sparse [25]. It is obvious that, in particular, patients with contraindications against systemic antifungals could benefit from such a multimodal approach.

Here, we retrospectively analyzed clinical improvement and healing by assessing the records of 56 patients with onychomycosis of the toenails that were treated in our clinic with laser therapy alone or with laser therapy in combination with topical antifungals.

## Patients and methods

In our retrospective analysis, we evaluated the records of all patients with proven onychomycosis caused by dermatophytes and affecting the toenails, including at least one hallux, which we treated with laser therapy in the time period of July 2013–December 2016. All patients enrolled in this study had a histologically and microbiologically proven diagnosis of onychomycosis caused by dermatophytes. We identified a total of 56 patients that fulfilled these criteria and included them in our analysis. In total, 27 patients received laser treatment alone and 29 patients received laser treatment plus topical antifungals (ciclopirox, or amorolfine nail lacquer or cream as indicated by the manufacturers).

The mean patient age was 62.4 years; 37 males (mean age = 64 years; age range = 27–92 years) and 19 females (mean age = 59 years, age range = 25–76 years) were included. Out of these 56 patients, 29 had already used topical antifungals (amorolfine or ciclopiroxolamine) for over 24 weeks and had reported that they did not observe any significant improvement prior to laser therapy. To standardize our analysis, we focused only on the toenail of one affected hallux. In all cases, fungal culture was positive for dermatophytes. In total, patients received an average rate of 3.9 laser sessions (range = 1–11). During every visit clinical courses were documented by standardized photography.

Treatment sessions were conducted every 2 weeks for the first 3 sessions and every 6 weeks thereafter. Laser treatment was performed with a 1.064-nm diode laser (FOX, A.R.C. Laser GmbH, Nuremberg) in pulsed mode with a spot size of 4 mm. Additional laser settings were power =  8 W, pulse duration = 80 ms, and repetition rate = 5.6 Hz. A total energy of 500–800 J was applied per session and great toenail depending on the treatment-associated pain. Prior to each laser session visible dystrophic nail material was mechanically removed by a podiatrist. During treatment, the laser beam was applied continuously with an average swipe speed of around 2–4 mm/s over the entire area of the nail and nail bed in a grid pattern until the patient reported moderate to strong heat.

### Assessment of therapeutic endpoints

Clinical and microbiological endpoints were systematically assessed. Patients’ and physicians’ evaluation of the therapeutic response was collected from the patients’ records. In addition, an independent dermatologist evaluated all photographs that had been taken before every session and at the final visit.

The primary endpoint, clinical improvement, was defined as a significant clinical improvement evaluated by the patient himself and assessment of the photographic documentation by an independent physician. Clinical cure was defined as 100% clear nail with no subjectable clinical signs of fungal nail infection via assessment of the photographic documentation by the independent physician. Furthermore, at the end of the last laser treatment, a final fungal culture and microscopic assessment of mechanically removed nail material were performed. Secondary endpoints were accordingly defined as the clearance of fungal pathogens in (i) microscopic examination and (ii) in fungal culture after up to 4 weeks of incubation. Direct fluorescence microscopy assay was used for microscopic examination. Therefore, Blankophor was used as a fast fluorescent whitener and direct detection of fungal elements was determined using a Zeiss Axioplan fluorescence microscope.

Fungal growth and spore production in culture medium were performed on Sabouraud agar for up to 3 weeks at room temperature. Mycological cure was defined as both negative microscopy and negative culture.

## Results

A total of 56 patients were treated with a 1.064-nm diode laser (FOX, A.R.C. Laser GmbH, Nuremberg) in pulse mode with a total energy of 500–800 J per nail of the hallux, depending on pain intensity.

### Number of treatments

The average number of treatments was 3.9 (range 1–11). Eleven patients received 1 laser session, 22 patients received 2–4 sessions, and 23 patients received more than 5 sessions.

### Clinical results

**Fig. 1:**
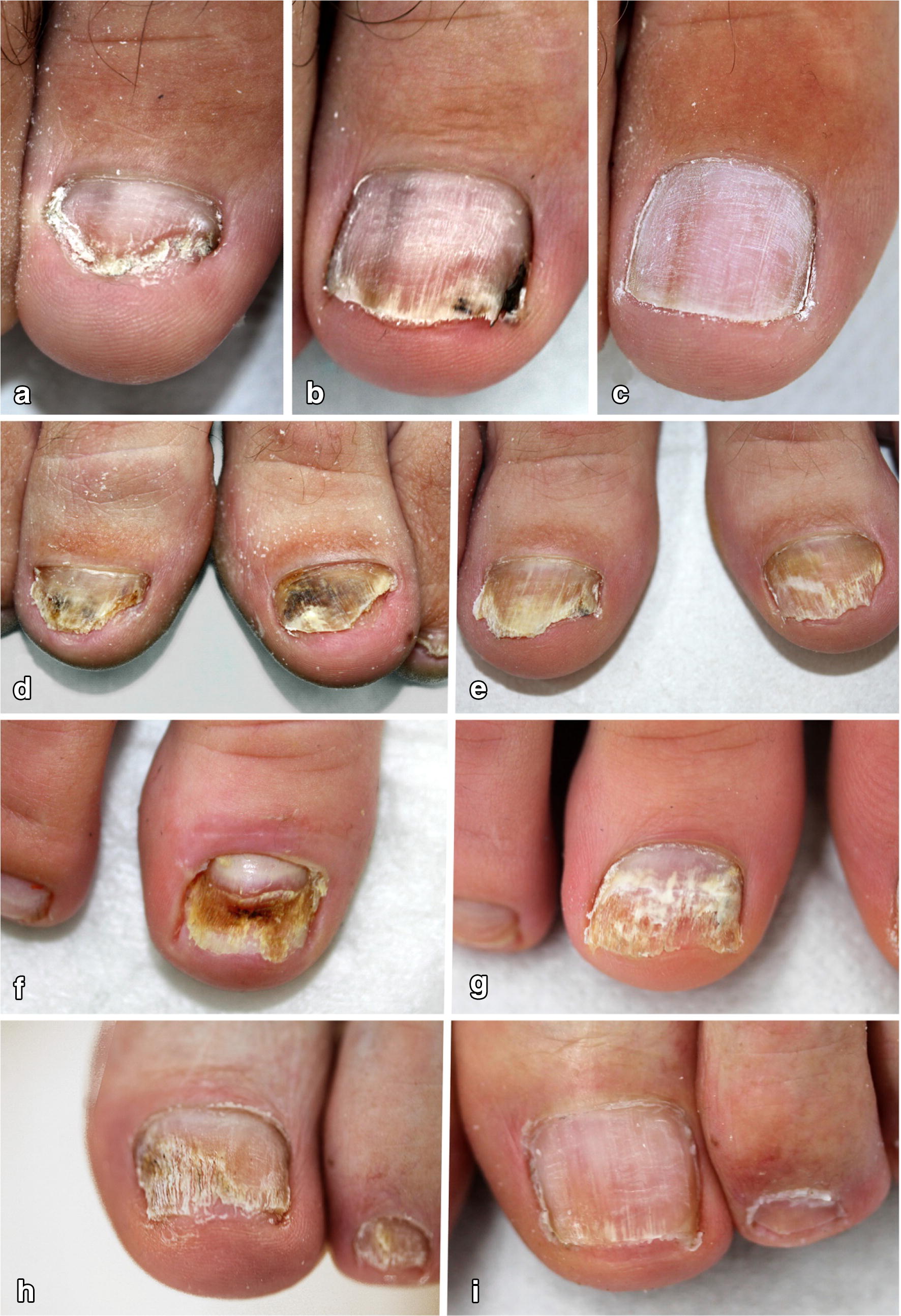
Clinical courses of hallux toenails treated with laser only or laser plus topical antifungals. Displayed are representative images of four patients before (left panel: **a**, **d**, **f**, **h**) and after (right panel: **c**, **f**, **g**, **i**) treatment with a 1.064-nm diode laser only (**a**–**c**) or laser plus topical antifungals (**d**–**i**). Individual representative courses show **b** clinical improvement after 36 weeks and 6 sessions and **c** complete cure after 68 weeks and 11 sessions with laser only (negative culture and microscopy); **e** clinical improvement after 30 weeks and 5 sessions of laser plus topical antifungals (negative culture and microscopy); **g** clinical improvement after 20 weeks and 5 sessions of laser plus topical antifungals (negative culture, positive microscopy); **i** clinical improvement after 24 weeks and 6 sessions of laser plus topical antifungals (negative culture, positive microscopy)

Table 1 shows the clinical results of the patients separated into groups. Figure 1 shows representative clinical courses. Patients treated with laser only received an average of 4.5 treatments. Patients treated with combination therapy received an average of 3.6 treatments. Significant subjective clinical improvement was achieved in 56% (*n* = 15) treated only with laser after 5.1 laser treatments, and in 69% (*n* = 20) treated in average 4.5 times with laser plus topical antifungals. For nails treated with laser only, complete clinical clearance was achieved in 26% as compared to 21% for nails treated with laser plus topical antifungals. For nails treated with laser plus topical antifungals, this result was achieved after an average 4.3 laser sessions as compared to 6.0 sessions for laser only. During therapy, besides mild pain and reported heat, no significant side effects were observed.

**Table 1:** Clinical efficacy of laser plus/minus topical antifungals for the treatment of onychomycosis

	Average of laser sessions (all patients)	Clinical improvement	Average of laser sessions	Clinical cure	Average of laser sessions
All patients *n* = 56	3.9	63% (*n* = 35)	4.9	23% (*n* = 13)	5.2
Laser only *n* = 27	4.5	56% (*n* = 15)	5.1	26% (*n* = 7)	6.0
Laser plus topical antifungals *n* = 29	3.6	69% (*n* = 20)	4.5	21% (*n* = 6)	4.3

### Mycological results

Table 2 shows the mycological results of the fungal culture. Table 3 shows the microscopic outcomes. The secondary endpoint “cultural cure” with no evidence of fungal growth in culture could be achieved in 75% (*n* = 42) of all patients. “Microscopic cure” (complete cure) was achieved in 16% (*n* = 9) of all patients.

**Table 2:** Cultural clearance after laser plus/minus topical antifungals for the treatment of onychomycosis

	Pretherapeutic positive	Posttherapeutic positive	Average of laser sessions
*n* = 56	100% (*n* = 56)	25% (*n* = 14)	4.9
Laser only *n* = 27	100% (*n* = 27)	37% (*n* = 10)	5.4
Laser plus topical antifungals *n* = 29	100% (*n* = 29)	14% (*n* = 4)	4.8

**Table 3:** Microscopic clearance after laser plus/minus topical antifungals for the treatment of onychomycosis

	Pretherapeutic positive	Posttherapeutic positive	Average of laser sessions
*n* = 56	100% (*n* = 56)	84% (*n* = 47)	4.2
Laser only *n* = 27	100% (*n* = 27)	89% (*n* = 24)	4.7
Laser plus topical antifungals *n* = 29	100% (*n* = 29)	79% (*n* = 23)	4

For patients treated with laser only, “cultural cure” was achieved in 63% after 5.4 laser sessions as compared to 86% of nails treated with laser plus topical antifungals after 4.8 laser sessions (Table 2). “Microscopic cure” (complete cure) was achieved in 11% of nails treated with laser only after 4.7 laser sessions, as compared to 21% of nails treated with laser plus topical antifungals after 4.0 sessions (Table 3).

## Discussion

The role of non-ablative laser therapy for the treatment of onychomycosis is discussed controversially. Published data and conclusions range from effective [25] to mostly ineffective [23, 24]. This heterogeneity of published data goes in line with a big heterogeneity in treatment protocols (wavelength and total energy applied, combination of treatments, number and duration of therapy) as well as methods of evaluation and assessed endpoints (e.g. physician or patient assessment, standardized photography, mycological cure, clinical improvement, clinical cure, and Onychomycosis Severity Index). This makes a direct comparison of results and the drawing of distinct conclusions difficult [15, 23, 25–34]. In particular, the clinical efficacy of multimodal approaches, applying laser and pharmacological therapies, remains ill defined.

Patients included in our analysis were treated with amorolfine or ciclopiroxolamine, lacquers or ointments. Amorolfine is a topical antifungal that inhibits Delta14-sterol reductase and cholestenol Delta-isomerase, thereby depleting ergosterol and causing a permeability of the fungal cell membrane. In an open-label, randomized, multicenter and controlled study conducted on 71 onychomycotic patients, treatment with amorolfine 5% lacquer after chemical nail avulsion with 40% urea showed clinical cure in 10.3% after 24 weeks and in 11.9% after 48 weeks. Complete cure comprising both negative direct microscopy and negative culture was achieved in 12.7% of all treated nails after 36 weeks [35]. In our hands, a comparable rate of complete cure of 11% was achieved in patients treated with laser only. However, 21% of patients treated with topical antifungals plus laser therapy achieved complete cure, indicating significant, additive effects.

Ciclopiroxolamine (ciclopirox) is a synthetic hydroxypyridine. Unlike most antifungals currently available, ciclopirox does not affect sterol biosynthesis. Instead, it involves chelation of polyvalent cations (such as Fe3^+^) with inhibition of metal-dependent enzymes responsible for the degradation of toxic peroxides in the fungal cell [36]. In a trial involving a total of 71 onychomycotic patients, the application of ciclopirox 8% lacquer after chemical nail avulsion for 24 weeks led to a 17.4% and after 48 weeks to a 42% clinical cure rate in onychomycosis. After 36 weeks, negative direct microscopy and negative culture were achieved in 36.6% of all treated nails [35]. Whereas these rates of complete cure significantly exceed the results observed in our analysis, it must be noted that a relevant number of 29 patients treated with a combination of topical antifungals and laser therapy in our hands had undergone a pharmacologic antifungal therapy (topical amorolfine or ciclopiroxolamine) for 24 weeks or longer without observing any significant improvement prior to laser therapy. Hence, our collective of patients could be characterized as more challenging and less responsive to pharmacologic measures as compared to therapy-naïve patients assessed in respective pharmacologic studies.

Reported rates for complete cure rates achieved by oral antifungals range between 55 and 78%, as reported by a cumulative meta-analysis by Gupta and colleagues [37]. These include terbinafine 250 mg daily for 3–4 months with 76–78% cure, itraconazole 400 mg in a three- to four-pulse regimen with 63–75% cure, and continuous intake of itraconazole 200 mg with a cure rate of 59–63%. The intake of griseofulvin 500–1000 mg daily up to 18 months is associated with a cure rate of 55–60%, whereas fluconazole 150 mg weekly for 3–12 months achieves a cure in only 48–53% [37]. Yet, even though these rates exceed results achieved for topical antifungals, lasers or combinations thereof, it is obvious that systemic therapy does not guarantee healing and, as noted before, in the daily practice systemic therapy is limited by contraindications, adverse events or simply refusal by patients [38–41].

With regard to the efficacy of laser therapy, in 2014, Raulin et al. performed a single-blind study comparing laser treatment with a 1064-nm long-pulsed and a short-pulsed Nd:YAG laser on onychomycotic toenails. At 9-month follow-up after two laser sessions the rate of complete cure in both groups was 20%. Negative fungal culture could be reached in 70% with the short-pulsed laser, compared with 60% with the long-pulsed laser [42]. Surprisingly, in a study performed by the same group in 2017, after 12 months no mycological, microscopic or even cosmetic improvement was reported for 82 onychomycotic nails treated four times with a short-pulsed 1064-nm Nd:YAG laser at intervals of 4–6 weeks [24]. The 1064-nm diode laser systems assessed in our analysis were also used in a clinical study performed by Renner and colleagues. Herein, 82 onychomycotic nails were treated with the respective 1064-nm diode laser as well as in combination with topical antifungals. Clinical efficacy was assessed using the Onychomycosis Severity Index (OSI) and showed an improvement of 25%. The treatment protocol did not include microscopic or cultural analyses of fungal pathogens [25].

Our results presented here show that treatment of onychomycosis using laser only does indeed have an effect, with a cultural cure rate of up to 63% (Table 2). This efficacy could be, furthermore, improved in combination with topical antifungals achieving up to 86% of cultural cure. Also patients that did not benefit from a prior topical pharmacologic antifungal therapy showed an improvement in combination with laser therapy. Surprisingly, 26% (7 of 27) patients showed a clinical cure after six laser treatments with laser only, whereas laser treatment in combination with topical antifungals resulted in a lower percentage of cure of 21% (6 of 29 patients); yet, this result was achieved after only 4.3 laser treatments (Table 1). This observation suggests that the efficacy of laser therapy is positively correlated to the overall number of laser treatments, and hence the outcome of therapy can be improved by increasing the number of laser sessions. Accordingly, we advise our patients to undergo at least three laser sessions before the individual response is evaluated. If a clinical improvement has been achieved up to this point, a continuation of laser therapy can be suggested until complete cure or until no further improvement may be observed.

Our results also show that out of a total of 56 patients 29 patients had been applying topical antifungals for 24 weeks or longer prior to the start of laser therapy without any significant effect. In combination with laser therapy eventually 69% of these 29 patients achieved clinical improvement and 21% achieved a complete clinical cure. While this effect could also be attributed to the sole continuation of pharmacologic measures, the timely correlation to the start of laser therapy suggests that mycological cure in these cases is indeed rather the result of combination therapy. Adverse effects of laser therapy for onychomycosis reported in the literature range from pain up to tissue necrosis [24, 43]. However, if performed responsibly it can be considered as safe and effective, as highlighted by our experience presented here. Finally, we can state from our experience that positive clinical results strongly correlated with the patients’ overall satisfaction with the treatment.

## Conclusions

Our results show that the application of a 1.064-nm diode laser is effective for the treatment of onychomycosis caused by dermatophytes achieving complete cure in 11% of the patients. Treatment efficacy can be, furthermore, increased by combining laser therapy with topical antifungals reaching up to 21% of complete cure. This multimodal approach is of particular interest for patients that are limited by contraindications against systemic antifungals. If performed responsibly laser therapy causes minimal to no side effects and comes along with a high patient satisfaction. In our hands, the overall efficacy of the therapy positively correlated with the total number of laser sessions.

Prospective, ideally intra-individually half-side controlled trials with relevant numbers of patients are still urgently needed to establish better treatment protocols and to support the proposed relevance of a multimodal, pharmaco-physical approach for the management of onychomycosis.
